# Structure and factorial invariance of a brief version of the Eating Attitudes Test in Peruvian university students

**DOI:** 10.3389/fpsyg.2023.1238211

**Published:** 2023-12-07

**Authors:** Cristian Ramos-Vera, Miguel Basauri-Delgado, Stefanny Huamán Obregón, Jacksaint Saintila

**Affiliations:** ^1^Área de Investigación, Universidad César Vallejo, Lima, Peru; ^2^Escuela de Postgrado, Universidad Femenina Sagrado Corazón, Lima, Peru; ^3^Universidad Autónoma del Perú, Lima, Peru; ^4^Escuela de Medicina Humana, Universidad Peruana Unión, Lima, Peru

**Keywords:** eating disorders, validity, reliability, factorial invariance, university students

## Abstract

**Background:**

University students often experience significant changes in their eating habits, which can increase the risk of developing eating disorders (ED). This situation calls for the creation of brief assessment tools to identify college students who may be most at risk. The aim of the study was to determine the psychometric properties of the Eating Attitudes Test-8 (EAT-8) in a Peruvian university population; additionally, the possible differences in the scores of the instrument according to sociodemographic variables, such as gender and age, were examined.

**Methods:**

A psychometric study was conducted on 610 participants (*M* = 24.3, SD = 2.16, and 61.5% female), aged 19 to 31 years, belonging to four universities of different professional careers.

**Results:**

The unidimensional eight-item model was found to have fit indices that confirm acceptable factorial validity (*X*^2^/df = 3.23, CFI = 0.984, TLI = 0.977, RMSEA = 0.061, SRMR = 0.049) and an internal consistency of 0.833 for the Cronbach's alpha coefficient and 0.838 for the McDonald's omega coefficient. In addition, the EAT-8 was reported to be invariant according to gender and age; likewise, there were no significant differences in the age and gender categories.

**Conclusion:**

The EAT-8 has solid psychometric properties, including validity, reliability, and invariance, in the Peruvian university population, which supports its ability to assess the risk of developing ED in this specific group.

## Introduction

University students often face several significant changes during their transition to university life. This period involves adapting to new routines, facing psychosocial changes, and dealing with the academic demands of higher education (Angelucci et al., 2017), which, in turn, can lead to changes in eating habits. In fact, university students are exposed to risk factors that may contribute to the development of Eating Disorders (EDs) due to lack of time, availability of unhealthy foods in the university environment, and inadequate dietary practices, which influence the adoption of unbalanced eating habits (Castelao-Naval et al., 2019). In addition, stress is constant in university life. High academic expectations, deadlines, exams, and other demands can generate high levels of stress in students. Stress can have a negative impact on eating patterns, either causing a decrease or increase in appetite, and in some cases, can trigger disordered eating behaviors (Castelao-Naval et al., 2019).

The EDs, such as anorexia nervosa (AN), bulimia nervosa (BN) and binge eating disorder (BED), are disorders that involve eating disorders such as intake restrictions, intense fear of weight gain and alterations in body image (Benítez et al., 2019). It is common that they start in adolescence, the most frequent being AN characterized by restriction of food intake with the purpose of achieving a weight and body shape considered ideal (Van Hoeken et al., 2016); therefore, they are diseases that cause medical complications and/or deaths.

BN is defined by the presence of recurrent episodes of binge eating, accompanied by a sense of lack of control, followed by inappropriate compensatory behaviors, such as vomiting or laxative use. On the other hand, BED is characterized by episodes of frequent binge eating, experienced with impulsivity and emotional distress, but without the presence of compensatory behaviors such as excessive exercise, vomiting or the use of laxatives (American Psychiatric Association, 2004). In Europe, a prevalence of 2.2% of ED has been found, while in America the prevalence is 4.6% and in Asia 3.5% (Arija-Val et al., 2022). Moreover, in the context of university students, the prevalence rate of ED is not accurate (Chinchilla, 2015). However, studies have been conducted in Ecuador indicating a prevalence of 14.5% (Pacherres, 2018), while 14% has been reported in India and Malaysia (Chan et al., 2020; Iyer and Shriraam, 2021). In Saudi Arabia, the prevalence is even higher at 32.5% (Abd El-Azeem Taha et al., 2018). In the case of Peru, it has been found that two out of 10 students are at risk of developing an ED (Zila-Velasque et al., 2022), possibly influenced by the country’s cultural diversity that shapes perspectives on body image, the increasing pressure of modern beauty standards in urbanized areas, and limited access to mental health education in various regions. In addition, the rapid urbanization and westernization of major Peruvian cities have led to changes in dietary patterns and exposure to global esthetic ideals, which, together with sociocultural factors, such as family and social pressure, may contribute to the increased risk of these disorders among young people.

Given the need to identify the early detection of risk factors that affect eating and thus prevent ED, multiple instruments have been developed to measure this construct, such as the *Questionnaire of Eating and Weight Patterns-Revised* (QEWP-R) (Spitzer et al., 1992); the *Eating Disorder Examination Questionnaire* (EDE-Q) (Fairburn and Beglin, 1994), the *SCOFF Test* (Morgan et al., 1999), and the *Eating Disorder Inventory* (EDI-2) (Garner, 1998). These instruments are the best known in the scientific community; however, the *Eating Attitudes Test* (EAT-26) (Garner et al., 1982) is a scale that measures risky eating attitudes and behaviors and has been applied in different countries such as Colombia (Constaín et al., 2014), Mexico (Lugo Salazar and Pineda, 2019), Spain, among others (Mu, 2004).

This instrument was developed by Garner and Garfinkel (1979), who initially considered 40 items (EAT-40); however, later, Garner et al. (1982) analyzed the psychometric properties of the instrument and confirmed the multidimensional structure of 26 items (EAT-26), which met the psychometric parameters (factor loadings >0.40) with adequate internal consistency values of the overall scale. According to the theoretical model, attitudes and behaviors related to ED are influenced by several factors. These include cultural factors, such as the influence of advertising, fashion, and the beauty industry, which promote certain standards of beauty and thinness. Family factors also play an important role, as the family environment can contribute significantly to the development of ED. In addition, there are perpetuating factors, such as social maladjustment, depression, and low self-esteem, which can maintain and aggravate ED. These factors interact in a complex way and may increase the risk of developing eating disorders (Garner, 1998).

In a study by McLean et al. (2023), inconsistencies were found in the 26-item model used in the measurement of eating disorders. The results of the confirmatory factor analysis showed that this model did not adequately fit the data obtained from the participants, with inadequate fit indices (CFI = 0.72, RMSEA = 0.11, GFI = 0.76, NFI = 0.67, TLI = 0.70). As an alternative, the researchers proposed a new factor model with 16 items, which proved to have better fit indices (CFI = 0.92; RMSEA = 0.09; GFI = 0.90; NFI = 0.90; TLI = 0.90). Similarly, Douka et al. (2009), when conducting the Confirmatory Factor Analysis (CFA) of EAT-26 in 167 university students, obtained low goodness-of-fit indices, where 13 items with low factor loadings were reported, which were eliminated to report a more optimal factor structure that meets the psychometric criteria (CFI = 0.97, SRMR = 0.06, RMSEA = 0.04). In addition, several psychometric studies of the instrument have reported inconsistencies in the number of dimensions of the EAT-26. In many of these studies, items have been eliminated to improve the structure of the questionnaire. Examples of these studies include research by Fekih-Romdhane et al. (2022), Haddad et al. (2021), Khaled et al. (2018), and Rogoza et al. (2016), who found the need to remove items from the EAT-26 in different samples. Moreover, specific studies have been conducted in a university population, such as those carried out by Ahmadi et al. (2014) and Lugo Salazar and Pineda (2019), who also identified the need to eliminate items from the questionnaire to obtain a more coherent and valid structure.

Due to the inconsistencies and limitations identified in the EAT-26, shorter versions of the instrument have been developed and validated. Some of these versions include EAT-18, EAT-7, and EAT-8. In Europe, the short 8-item version of the Eating Attitudes Test (EAT-8) has been validated in a sample of 2,527 participants, aged between 14 and 95 years (Richter et al., 2016). This brief version has presented adequate goodness-of-fit indices (RMSEA = 0.035, CFI = 0.995, TLI = 0.993). Regarding convergent validity, scores on the EAT-8 correlated significantly with scores on the *SCOFF screening* instrument (*r* = 0.43) and the *ICD-10-Symptom-Rating* (ISR-E) (Tritt et al., 2008) that measures psychological symptoms (*r* = 0.59). The bivariate correlation between lifetime self-diagnosis of ED and EAT-8 scores revealed a low positive correlation (*r* = 0.13), compared to other studies conducted in various settings. An example of validation of the EAT-8 in Asia is the study conducted by Asl et al. (2020) on a sample of 302 students, where both the internal consistency and CFA of the EAT-8 were evaluated; adequate internal consistency was reported and the goodness-of-fit indices of the unidimensional model in the CFA were also considered acceptable (RMSEA = 0.08, CFI = 0.97, SRMR = 0.04); in addition, convergent validity was presented with the scores of the self-report measures of the *Eating Beliefs Questionnaire* (EBQ-18), an instrument that estimates fear of food.

In a study of Spanish adults, it was evidenced that men have a more realistic perception of their body, while women tend to overestimate it (Soto Ruiz et al., 2015), as they report greater concern and dissatisfaction with their weight and body image (Escolar et al., 2017). A study conducted in Peru by Zila-Velasque et al. (2022) found that female university students from 22 universities have an increased risk of developing eating disorders compared to males. This finding is consistent with a systematic review by Lindvall Dahlgren et al. (2017), which included 19 articles and a total of 22,397 participants from 10 countries. The difference in the prevalence of ED between female and male individuals can be attributed to several factors. Studies, such as Vijayalakshmi et al. (2017), have noted that females tend to have a greater inclination toward thinness, a greater propensity to engage in disordered eating behaviors, and greater body dissatisfaction compared to males.

In turn, in terms of age, a systematic review of articles from Europe, Asia, and America found that young women are at the highest risk of developing an ED (Arija-Val et al., 2022). These results have also been evidenced in studies that have been carried out in a university population. For example, a study conducted in Spanish students found a negative correlation between age and EAT-8 total score, suggesting that younger participants (in the range of 14–29 years) presented slightly higher scores on the scale (Richter et al., 2016). An additional study conducted in university students revealed that there are significant differences in terms of risk symptomatology for the development of eating disorders in those younger students, specifically those under 22 years of age (Escolar et al., 2017).

Factor invariance analysis is of utmost importance in studies that seek to compare two or more groups in terms of variables such as age, gender, or educational level. This analysis ensures that the differences observed between groups are genuine in relation to the construct being assessed and are not simply due to different responses to the items (Byrne, 2008). Therefore, this study examines the measurement equivalence of EAT-8 to identify possible differences as a function of age and gender. This is important to avoid biased interpretations of differences that may be due to a lack of measurement invariance (Byrne, 2008). So far, only three previous studies have been conducted that have examined the invariance of EAT-26 in university students (Richter et al., 2016; Torres et al., 2017). However, there is no specific research that has addressed the invariance of EAT-8 in this population. Therefore, this study seeks to fill that gap and provide additional information on the reliability and validity of the EAT-8 in university students.

A review of the scientific literature reveals a lack of research on the psychometric properties of EAT-8 in Peruvian university students. Therefore, a validated and reliable instrument for the clinical practice of mental health professionals is totally useful and relevant, in addition to being a novel contribution to the scientific community. Additionally, by employing a network-based methodological approach, it is possible to graphically visualize the relationships between each EAT-8 indicator, providing insights that other statistical methods do not offer, such as understanding the role that a particular item plays within the network and how it may influence other indicators (Hevey, 2018). However, methodological differences in prevalence limit the comparison of data by age and gender; for this reason, studies are needed to assess the evolution of the prevalence of ED according to these factors. Therefore, the following objectives were proposed: (a) To determine the psychometric properties of the EAT-8 in the Peruvian university population; (b) To evaluate measurement invariance with respect to gender and age; (c) To identify the relationship between the items of the EAT-8, as well as their centrality indexes, by means of network analysis; and (d) To know the differences in this measure between the sociodemographic categories of gender and age.

## Materials and methods

### Study design

In this research, the psychometric properties of the EAT-8 scale were measured at a specific time and without deliberate manipulation of the variable; therefore, it was instrumental, non-experimental, and cross-sectional.

### Participants

The study consisted of 610 university students from Metropolitan Lima, aged 19–31 years (*M* = 24.3, SD = 2.16, and 61.5% female), and from middle socioeconomic levels. The careers to which they belonged were health sciences (430), engineering (61), social sciences and humanities (63), and business sciences (56). Age was grouped as follows: 19–24 years (48.6%) and 25 to 31 years (51.5%). As for the sampling process used in this study, a non-probabilistic method known as “snowball” was employed. This strategy consists of initially identifying an individual who meets the characteristics of interest in the population and asking him or her to refer other potential participants. In this way, a successive chain of referred participants is generated, allowing the sample to be expanded until the desired size is reached. In this study, certain inclusion criteria were established to select participants. These criteria included being university students enrolled during the 2020–2021 period, being older adults (18 years and older), being older adults, being students from four universities located in Lima, having given informed consent to participate in the study, and having Peruvian nationality.

### Instrument

#### Eating attitudes test

This instrument was constructed by Garner and Garfinkel (1979). The Spanish version of Mu (2004) was used. A brief 8-item version of Richter et al. (2016) was validated by obtaining adequate psychometric properties. This instrument measures the risk of suffering any type of ED in women and men. This brief version is unidimensional and consists of 8 Likert-type items: always (Arija-Val et al., 2022), almost always (American Psychiatric Association, 2004), frequently (Van Hoeken et al., 2016), sometimes (Benítez et al., 2019), rarely (Castelao-Naval et al., 2019), and never (Angelucci et al., 2017). In terms of scoring, all responses for each item are summed, where a higher score refers to a higher risk of developing an ED. Regarding the psychometric properties of the version used, it presents concurrent validity with a measure of eating disorder syndrome (*r* = 0.59; ISR-E) and core symptoms of anorexia and bulimia nervosa (*r* = 0.43; SCOFF). Validity was calculated with the CFA and acceptable fit indices were obtained for the unidimensional model (*X*^2^ = 82.55, df = 20, CFI = 0.995, TLI = 0.993, RMSEA = 0.035). The reliability for this version was 0.83 (Richter et al., 2016).

### Procedure

This research was conducted with the approval of the Ethics Committee of the Universidad Autónoma del Perú. Due to the circumstances of the COVID-19 pandemic, during the month of January to March 2020. The administration of EAT-8 was conducted virtually using the Google Forms platform. The application process followed the internet-based methodology, which includes compliance with established ethical recommendations, such as obtaining informed consent from participants. In addition, the ethical principles established in the Declaration of Helsinki and in the Code of Ethics and Deontology of the College of Psychologists of Peru were considered. These ethical guidelines were considered to ensure the protection of participants’ rights and compliance with ethical standards in the conduct of the research. The participants’ data were collected by distributing the link to the form through various social networks, such as WhatsApp, Facebook, and Gmail. The researchers shared the link with the people they had contact with, who in turn shared it with their acquaintances who also met the same characteristics. Data collection was conducted over a period from May through June 2020.

Initially, *N* = 620 Peruvian university students were recruited to participate in the study. However, 10 participants were eliminated because they did not meet all the required sociodemographic criteria. Finally, the sample consisted of 610 students. Upon accessing the online survey, informed consent was obtained; furthermore, on the home page, the inclusion criteria were mentioned as requirements to proceed to complete the subsequent sections. These sections included the collection of sociodemographic data and the application of EAT-8. All information collected was kept anonymous and confidential because the ethical principles of beneficence, nonmaleficence, justice and respect of the Declaration of Helsinki were complied with.

### Data analysis

All statistical analyses (descriptive, confirmatory factor analysis, reliability, invariance, network analysis, and Bayesian difference) were performed with the freely available software Jeffrey’s Amazing Statistics Program (JASP) version 0.17.1.

The data analysis consisted of six stages. In the first, the descriptive results for each of the items were recognized, where values such as mean, standard deviation, skewness, and kurtosis were reported. These two analyses provided information on univariate normality by verifying that the scores were within ±1.5 standard deviations (Hancock et al., 2010). Additionally, the item-test correlation (Rit) was also calculated for the eight items to examine multicollinearity, looking for values above 0.30 (Hagell and Westergren, 2006).

In the second stage, CFA was used to determine the validity based on the internal structure of the EAT-8. For such analysis, the Weighted Least Square Mean and Variance adjusted (WLSMV) was considered as estimator because the data were ordinal (Gana and Broc, 2019). To identify the proposed model as valid, the following fit indices were considered: Chi-square to degrees of freedom ratio (*X*^2^/df < 4) (Hair et al., 2014), comparative fit index (CFI > 0.90), Turkes-Lewis index (TLI > 0.90), root mean square error of approximation (RMSEA ≤ 0.08), and standardized root mean square residual (SRMR ≤ 0.08) (Hu and Bentler, 1999). Additionally, items with saturations above 0.30 were accepted (Herrero, 2010). While in the third stage, internal consistency reliability was calculated by means of the Cronbach’s alpha (α) and McDonald’s omega (ω) coefficients, which are considered acceptable when reaching values above 0.70 (Viladrich et al., 2017).

In the fourth stage, a multigroup CFA (MG-CFA) was performed with the unidimensional model to examine invariance as a function of gender and age. This analysis allows us to evaluate the constancy of the factorial structure in the different groups, i.e., it tries to establish whether the patterns of each factorial loading are the same among the groupings, which is fundamental for making valid comparisons (Dimitrov, 2010). Initially, configural invariance was reviewed as a baseline measure, then constraints were progressively added to calculate the other invariance models (metric, scalar, and residual). Therefore, to consider a measurement model as invariant, the following values were taken into account: ΔCFI = 0.01, ΔRMSEA and ΔSRMR = 0.015 (Byrne, 2008).

In the fifth stage of the study, a psychometric network analysis was performed, which is a useful tool for examining the partial relationships between variables in the form of positive or negative edges connecting different nodes. This approach makes it possible to visualize and understand the structure and dynamics of the relationships between the variables studied. In the current study, each of the EAT-8 indicators was considered as a specific node within the analyzed network. The EBICglasso estimator was used in the analysis, since this technique facilitates the identification of the most significant connections between the nodes or indicators (Foygel and Drton, 2010). In addition, centrality indices (betweenness, closeness, and strength) were evaluated to estimate the interconnectedness in the network structure (Opsahl et al., 2010). The indicator of higher centrality would present a stronger influence on the other network traits (Epskamp et al., 2018; Ramos-Vera, 2021). These indices are the degree of connectivity (betweenness centrality), the proximity between all variables in the network (closeness centrality), and the frequency of connections that each node has to the number of possible connections (strength centrality) (Ramos-Vera and Serpa, 2021; Ramos-Vera et al., 2023).

In the final stage of the study, the Mann–Whitney Bayesian U test was used to identify possible differences according to sex and age in relation to the overall EAT-8 score. This statistic was used because the normality test indicated the use of nonparametric statistics (*p* < 0.05). The results identify the Bayes Factor (BF), which has two interpretations: BF10 (in favor of the alternative hypothesis of significant difference) and BF01 (in favor of the null hypothesis of significant difference), along with the 95% confidence intervals and the effect size (Ly et al., 2018). The evidentiary strength of the significance hypotheses was calculated using the Jeffreys criteria for comparative studies: weak, moderate, strong, very strong, and extreme (Ramos-Vera et al., 2021; Ramos-Vera, 2022).

All statistical analyses (descriptive, confirmatory factor analysis, reliability, invariance, network analysis, and Bayesian difference) were performed with the freely available software Jeffrey’s Amazing Statistics Program (JASP) version 0.17.1.

## Results

### Descriptive

Table 1 shows a higher score for items 3 (*M* = 1.184 and SD = 0.948) and 5 (*M* = 1.174 and SD = 0.880), while the mean for the total scale was 6.822 (SD = 4.650). In addition, there is univariate normality because skewness and kurtosis were found within the criteria of +1.5 to −1.5 (Bandalos and Finney, 2019). The item - test correlation was 0.446–0.638, higher than the minimum value of 0.30 (Hagell and Westergren, 2006).

**Table 1:** EAT-8 descriptives.

Items	*M*	SD	g1	g2	Rit
Item 1	0.679	0.791	1.137	0.969	0.446
Item 2	0.557	0.831	1.497	1.525	0.587
Item 3	1.184	0.948	0.276	−0.914	0.502
Item 4	0.643	0.892	1.336	0.908	0.635
Item 5	1.131	0.876	0.624	−0.156	0.638
Item 6	1.174	0.880	0.568	−0.258	0.606
Item 7	0.641	0.788	1.173	0.933	0.375
Item 8	0.813	0.825	0.781	−0.198	0.343
EAT-8 (total)	6.822	4.650	0.956	0.885	–

M, mean; SD, standard deviation; g1, skewness; g2, kurtosis; Rit, item-test correlation.

### Factorial validity

The AFC of EAT-8 showed that the fit indices were within considerable parameters to confirm the unidimensional model (*X*^2^/df = 3.23, CFI = 0.984, TLI = 0.977, RMSEA = 0.061, SRMR = 0.049). In addition, factor loadings ranged from 0.384 to 0.779 (Table 2), which allowed the retention of all items in the model given that the saturations were above 0.30 (Herrero, 2010). For this reason, it is considered that the EAT-8 has evidence of validity based on internal structure.

**Table 2:** Factor loadings of EAT-8I.

Items	λ	*p*
1. Consumo pocos alimentos dietéticos (light)/I eat diet foods	0.384	<0.001
2. Me siento demasiado culpable después de comer/I feel extremely guilty after eating	0.779	<0.001
3. Solo pienso en quemar calorías cuando hago ejercicios/I think about burning up calories when I exercise	0.534	<0.001
4. Me siento culpable e incómodo después de haber comido dulces/I feel uncomfortable after eating sweets	0.746	<0.001
5. Me preocupo por estar más delgado y poder llegar a mi peso ideal/I find myself preoccupied with food	0.723	<0.001
6. Me preocupa la idea de tener grasa en el cuerpo/I am terrified about being overweight	0.680	<0.001
7. Me encuentro preocupado por la comida/7 I am preoccupied with a desire to be thinner	0.582	<0.001
8. Me aterroriza el sobrepeso/I am preoccupied with the thought of having fat on my body	0.541	<0.001

λ = factor loadings, p = statistical significance.

### Reliability

It was found that the questionnaire showed adequate reliability in terms of internal consistency. The Cronbach’s alpha coefficient (α) obtained was 0.833, while the McDonald’s omega coefficient (ω) yielded a satisfactory value of 0.838. These results suggest that the instrument used is reliable for measuring the construct of interest, because the scores obtained were above 0.70 (Viladrich et al., 2017).

### Factorial invariance

The equivalence analysis of the EAT-8 was performed by means of the AFC-MG, where the gender category was considered, and the factorial invariance models were progressively evaluated (Table 3) (Byrne, 2008). We initially reported a configural invariance with acceptable fit indices (CFI = 0.994, TLI = 0.992, RMSEA = 0.029, and SRMR = 0.054) that served as a baseline for comparison with the other models. Subsequently, the EAT-8 metric invariance model was found to meet the established criteria (ΔCFI ≤ 0.01 and ΔRMSEA and ΔSRMR ≤ 0.015) (Chen, 2007). In addition, scalar invariance (ΔCFI = 0.004, ΔRMSEA = 0.002, and ΔSRMR = 0.000) and residual invariance (CFI = 0.000, ΔRMSEA = 0.004 and ΔSRMR = 0.003) were verified. These results indicated that the questionnaire maintained the same measurement scale for both groups of men and women, reflecting fairness in the assessment of the constructs of interest. Similarly, the four invariance models were estimated in a stepwise manner according to age and acceptable differences were found (ΔCFI ≤ 0.01 and ΔRMSEA and ΔSRMR ≤ 0.015), which allowed us to consider that the EAT-8 is invariant between students aged 17–24 years and those aged 25–31 years.

**Table 3:** Factorial invariance of EAT-8.

	*X*^2^/df	CFI	TLI	RMSEA	SRMR	ΔCFI	ΔRMSEA	ΔSRMR
**Gender**								
Configural	1.25	0.994	0.992	0.029	0.054	–	–	–
Metric	1.74	0.991	0.987	0.039	0.066	0.003	0.010	0.012
Scalar	1.51	0.985	0.984	0.041	0.066	0.006	0.002	0.000
Residual	1.42	0.985	0.987	0.037	0.069	0.000	0.004	0.003
**Age**								
Configural	1.47	0.989	0.985	0.049	0.057	–	–	–
Metric	1.49	0.986	0.985	0.049	0.064	0.003	0.000	0.007
Scalar	1.25	0.992	0.992	0.035	0.064	0.006	0.014	0.000
Residual	1.06	0.998	0.998	0.027	0.064	0.006	0.008	0.000

X^2^/df, Chi-square degree of freedom; CFI, Comparative Fit Index; TLI, Tucker-Lewis Index; RMSEA, Root mean square error squared root; SRMR, Standardized root mean square residual; ΔCFI, Comparative fit index difference; ΔRMSEA, Root mean square error squared root differences; ΔSRMR, Standardized root mean square residual differences.

### Network analysis

**Figure 1:**
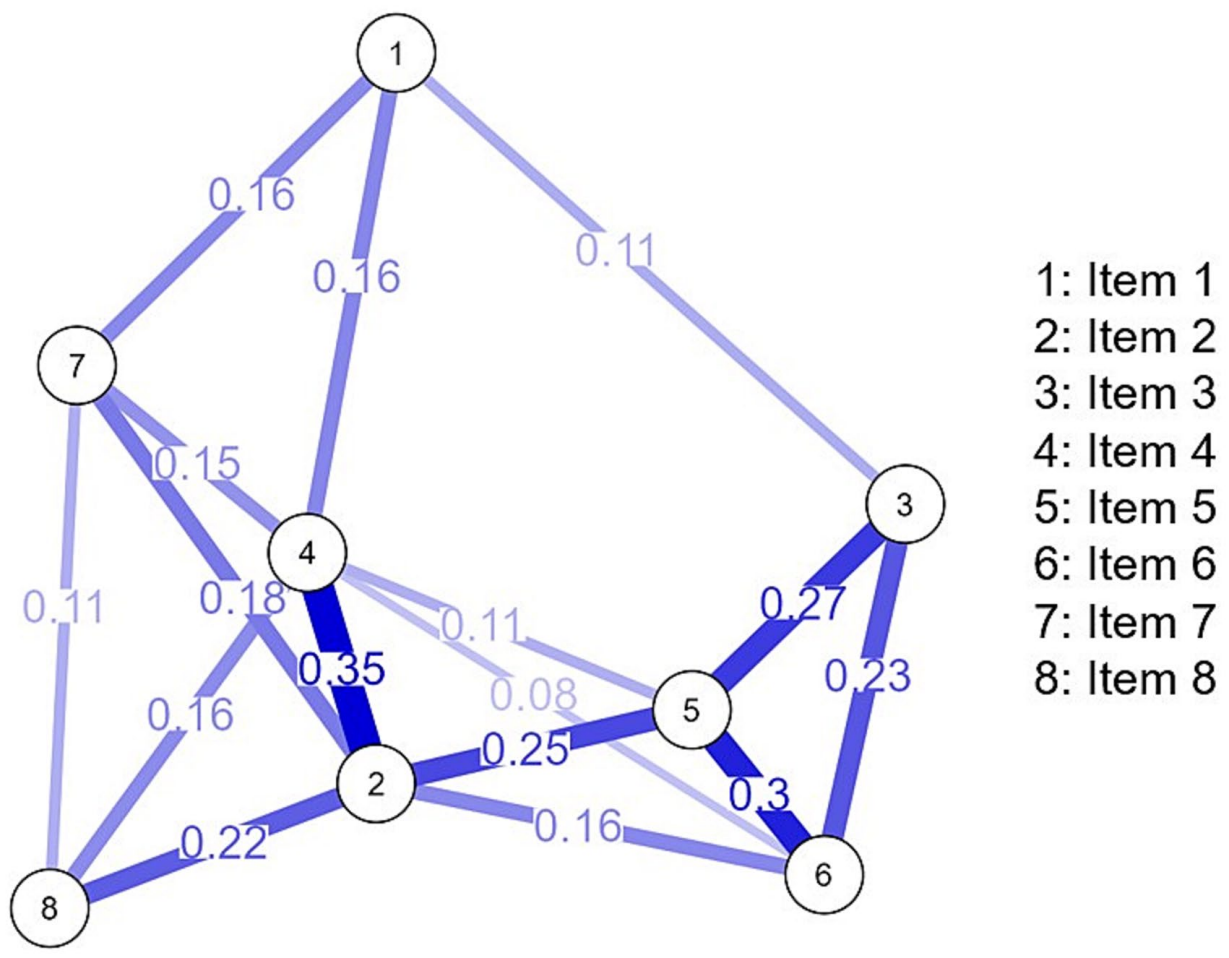
EAT-8 network analysis.

**Figure 2:**
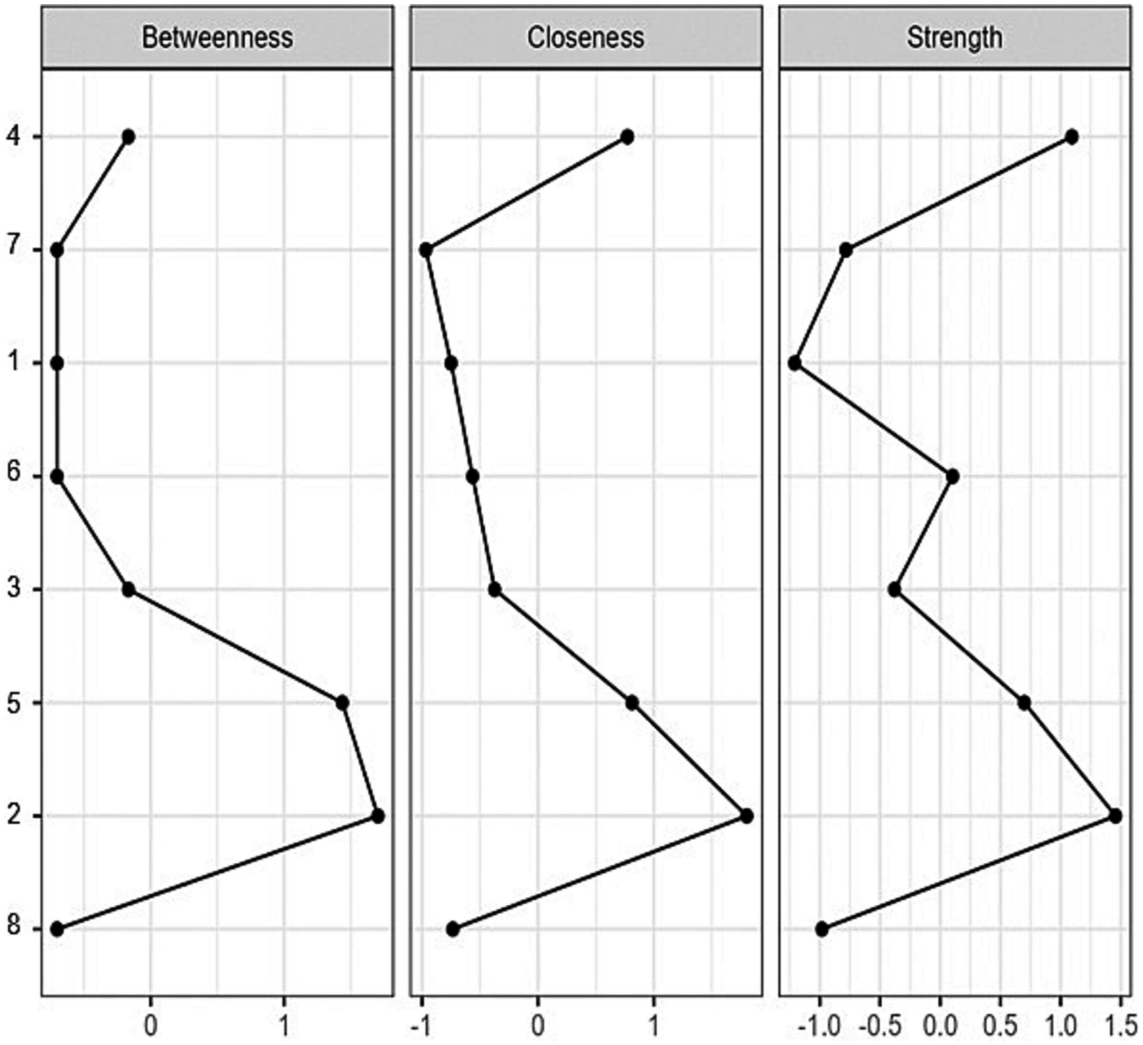
EAT-8 centrality indexes. Centrality indices are shown as standardized Z-scores.

Figure 1 shows that all items of EAT-8 presented at least three direct relationships, where the strongest was between items 2 and 4 (*r* = 0.35), 5 and 6 (*r* = 0.30) and 5 and 3 (*r* = 0.27). While the lowest association was between items 4 and 6 (*r* = 0.08). These results show that the partial correlations exhibit weak to moderate effect sizes (Epskamp et al., 2018). As for the centrality values (Figure 2), it was decided to consider the strength centrality because it allows us to recognize the item that has a greater interconnection influence on the other variables (Mcnally, 2021; Ramos-Vera, 2022). In the present network, it was recognized that item 2 (1.46) and item 4 (1.09) had the highest centrality scores, however, the indicator with the lowest strength was item 1 (−1.21).

### Differences according to gender

Using the Bayesian Mann U test, differences in the EAT-8 total score according to sex were identified. The probability strength in favor of the null statistical hypothesis was found to be moderate (Ramos-Vera, 2021), being nine times greater than the alternative hypothesis (BF10 = 0.101, BF01 = 9.934). In addition, a small estimated effect size was observed (effect = −0.033 [−0.195, 0.130]). Regarding the comparison according to age, the probability strength was found to be moderate and in favor of the null hypothesis, being three times greater than the alternative hypothesis (BF10 = 0.275, BF01 = 3.643). The estimated effect was of small size (effect = 0.120 [−0.042, 0.284]). The findings show that there are no significant differences according to gender and age for EAT-8.

## Discussion

Given the vulnerability of the university population to develop ED, several instruments have been developed to assess the construct. Some of these instruments include the QEWP-R, EDE-Q, SCOFF Test, and EAT, which have been used to measure and assess the presence of ED in this population group (Spitzer et al., 1992; Fairburn and Beglin, 1994; Garner, 1998). The EAT scale stands out among the aforementioned instruments due to its wide use and impact in several countries in the Americas, Asia, and Europe. Its popularity is due to its ability to effectively assess the presence of EDs in different cultural and geographical contexts (Mu, 2004; Douka et al., 2009; Richter et al., 2016; Rogoza et al., 2016; Lugo Salazar and Pineda, 2019). The evaluation of the psychometric properties of EAT-8 in university students is especially relevant due to the lack of research in the South American population. It can be noted that several countries in this continent are developing and maintain a strong influence of Western countries, where EAT rates tend to be higher (Kolar et al., 2016). Different studies report that in the urbanized regions of Latin America there are EBD levels like those found in the American and European populations (Palavras et al., 2011; Kessler et al., 2013). In addition, within Latin American culture, food has a high emotional value, which can be reflected in culinary traditions, food-related festivities and even in the language (for example, sayings such as “Barriga llena, corazón contento”/“Full belly, happy heart” or “Las penas con pan duelen menos”/“The sorrows with bread hurt less”) (Reyes-Rodríguez et al., 2016; Cevallos et al., 2023). Given that eating disorders tend to begin during adolescence and persist through youth and university, and considering that this specific population may be more exposed to risk factors and psychological comorbidities that may increase the likelihood of developing eating disorders, therefore, it is important to have a reliable and valid assessment tool to accurately and early identify the presence of these disorders in this population group in order to provide appropriate intervention and support.

CFA of the EAT-8 has shown that the fit indices are within the parameters established to support the unidimensional model. In addition, the items of the instrument show acceptable factor loadings above 0.30, which reinforces the validity based on the internal structure of EAT-8. The findings obtained in this study are consistent with previous research by Richter et al. (2016), who validated the EAT-8 version in a sample of 2,527 participants aged 14–95 years in Germany. Using the CFA, they found that the factor model of EAT-8 had adequate goodness-of-fit indices and optimal internal consistency (α = 0.85). In addition, the study by Asl et al. (2020) also provided consistent psychometric results using the AFC of EAT-8 in a sample of 302 students at Tehran University in Iran, evidencing findings that support the unidimensional model of EAT-8, showing acceptable factor loadings ranging from 0.67 to 0.82. These results further reinforce the internal structure and validity of the EAT-8 as a reliable instrument for assessing EDs in university settings.

Contrary to previous findings, a study conducted by McLean et al. (2023) pointed out inconsistencies in the adaptations of EAT-26. In their analysis conducted on a sample of 1,271 Australian participants, specifically vegetarians and vegans, they found that the proposed trifactorial model for EAT-26 did not present an adequate fit. In response to these findings, the researchers proposed an alternative multidimensional model consisting of 16 items. In another adaptation study by Homan et al. (2018), the adapted version of EAT-26 was also found to present an inadequate fit to the data. In a sample of 490 participants, it was identified that the originally proposed three-factor model was not appropriate. As a result, steps were taken to improve the fit of the model, and five items were removed from the scale. Other more recent psychometric studies support the lack of factorial consistency of EAT-26 in samples of university students in both the United States (Cesareo et al., 2018) and Russia (Meshkova et al., 2023). Both Meshkova et al. (2023) and Cesareo et al. (2018) found evidence suggesting the need to remove several items from the instrument to achieve an adequate fit to the instrument structure. These findings highlight the importance of making adaptations and refinements to assessment instruments to ensure their validity and usefulness in different contexts.

It is important to note that the two previous studies on EAT-8 conducted by Richter et al. (2016) and Asl et al. (2020) used Cronbach’s alpha coefficient exclusively, unlike the approach used in the present study. Precisely, in the current study, the McDonald’s omega coefficient was used to evaluate the reliability of the scale. This more up-to-date approach considers the factor loadings of the items, which provides a more stable estimate of the reliability of the latent variable. This approach has been supported by researchers such as Gerbing and Anderson (1988) and McDonald (1999).

Regarding the invariance according to gender, a configural invariance was found, with adequate adjustment indexes, which allowed progress in the evaluation of the metric, scalar, and residual invariance according to the established criteria. These results support the equity of measurement between men and women in EAT-8. The factorial invariance, in relation to age (19–24 years and 25–31 years), was clearly evidenced, and possible discrepancies are not the result of measurement bias. This study is the first to provide evidence of such a characteristic in relation to both demographic groups for the EAT-8. So far, only one other research has been reported evaluating the gender and age invariance of EAT-15 version (15 items) in a sample of 717 Iranian adolescents (Sahlan and Sala, 2022). In addition, according to the psychometric literature of this instrument, the equivalence of measurement between categorical groups according to sociodemographic aspects such as race, language, and religion is reported (Belon et al., 2011; Khaled et al., 2018; Spivak-Lavi et al., 2021). In relation to the university sample, a previous study has been conducted confirming the invariance according to sex and age of another measure of ED risk symptoms, specifically the Dutch Eating Behavior Questionnaire, in a sample of 990 Italian adults (Dakanalis et al., 2013). Therefore, our results add to the instrumental findings of psychopathological risk vulnerability to EDs, which confirm the equivalence between these categorical groups, specifically in Peruvian university students.

In the network analysis, it was identified that item 2 (“Me siento demasiado culpable después de comer”/I feel extremely guilty after eating) presents a direct relationship of greater intensity with item 4 (“Me siento culpable e incómodo(a) después de haber comido dulces”/I feel uncomfortable after eating sweets). These items also have a higher centrality (strength), i.e., they are the most influential indicators in the connection with the other symptoms of EAT-8. This specific association between the aforementioned items has also been observed in another recent study involving 1,203 participants from North America (Punzi et al., 2022). In addition, a higher degree of centrality (strength) for the initially mentioned measure of guilt feeling has been reported in four different networks that included adolescent and adult patients with AN and BN being treated in a hospital center in Germany (Schlegl et al., 2021). Furthermore, a network study by Sahlan et al. (2021) supports this finding in adolescents and young adults in Iran, who also presented symptoms of depression and anxiety in the network analysis. More recent research in Asian university students has also corroborated the central role of this item in network systems, which contributes to the presence of symptoms of increased psychopathological vulnerability in relation to eating behavior. In addition, a stronger association has been found with other symptoms of negative affect and poor resilient attitudes (Sahlan et al., 2022; Sahlan and Sala, 2023).

Item 2 (“Me siento demasiado culpable después de comer”/I feel extremely guilty after eating) has been shown to be the most relevant in the sample of university students and has been observed to be associated with low self-esteem and emotional problems (Mason et al., 2021). These associations are especially prominent in female students, who also show a tendency to reduce their body mass index (Monteleone et al., 2019). The measure in question shows a direct relationship with item 5 (“I worry about being thinner and being able to reach my ideal weight”). Both items present a greater interconnectedness in the network (high degree of betweenness and closeness), due to their location as central nodes and their proximity to other symptoms of vulnerability to eating disorders. In addition, these measures have been found to have greater connecting significance in other network studies conducted in samples of patients with eating disorders. For example, in a study by Elliott et al. (2020), it was found that having a greater sense of guilt about food and a greater concern about weight were associated with greater comorbidity of stress and depressive symptoms over three time periods (6, 12, and 24 months). These measures have also been highlighted in other network studies. For example, in a study by Calugi et al. (2021) with Italian participants diagnosed with AN, these measures were found to be central both before and after cognitive-behavioral psychotherapeutic treatment, suggesting that food guilt and weight preoccupation play an important role in the symptomatology of AN and continue to be relevant even after treatment. Another investigation that found similar findings was conducted in Italy by Calugi et al. (2020) and involved 214 individuals diagnosed with anorexia nervosa. Therefore, the preoccupation with staying thin is a high-risk attitude for the development of eating disorders, as it fosters the desire to have a thinner body figure (Rogoza et al., 2016). This concern in turn contributes to people experiencing feelings of guilt after eating (Forrest et al., 2019). These risk measures are especially related in individuals diagnosed with EDs who have elevated body mass indices (Monteleone et al., 2019). However, university students are not exempt from facing these issues, as they are exposed to social influences, especially through the media and social networks, which promote certain standards of beauty (Al Banna et al., 2021; Yao et al., 2021). These standards may increase concerns about physical appearance, body image, body weight, and beauty in university students. As a result, some students may develop eating-related problems, such as EDs. It is critical to address these risk factors in the prevention and treatment of EDs in this population group.

It was observed that item 4 (“Me siento culpable e incómodo después de comer dulces”/I feel uncomfortable after eating sweets) was one of the most prominent indicators within the internal structure of the EAT. This association has been supported by previous research using the instrument using a sample of 785 U.S. university students, as reported by Ocker et al. (2007). Similarly, this item had greater concordance and temporal stability in a sample of young women from southern Brazil (Nunes et al., 2005). Furthermore, in the Peruvian context, this information is supported, given that, due to the economic crisis, lack of time and abundance of fast food restaurants, university students choose to consume sugary foods at various times of the day and replace fruits or vegetables with breads or sweets (Villena, 2017). Accordingly, this finding suggests that the measure of feelings of guilt about eating sweets is of greater importance in a sample of university students. This is because this group presents a preference for foods and beverages with high sugar content, which exposes them to an increased risk of overweight and obesity, as observed in previous research conducted by Byrd-Bredbenner et al. (2012) and Nakhooda and Wiles (2018).

Another relationship of greater intensity was between item 5 (“Me preocupo por estar más delgado y poder llegar a mi peso ideal”/I find myself preoccupied with food) and item 6 (“Me preocupa la idea de tener grasa en el cuerpo”/I am terrified about being overweight), since both indicators allow us to recognize the concern about physical appearance, whose network relationship has also been reported in European participants from England, Spain, Austria, and Slovenia (Giles et al., 2022). This preoccupation with appearance in university students is intensified using social networks and can lead to emotional problems such as increased social anxiety and depression (Hawes et al., 2020). One consequence is the drive/desire to lose weight, which may arise to a greater extent in university students as they come to compare and evaluate themselves against visual representations of celebrities, peers, friends, or even strangers they perceive in their social networks (Saunders and Eaton, 2018; Anixiadis et al., 2019). In most network studies reviewed in two systematic reviews, concern and desire to lose weight were found to be the most prominent and central measures (Monteleone et al., 2019; Punzi et al., 2022).

Our comparative results indicated that no significant differences were found in EAT-8 scores according to gender, which is consistent with other studies conducted in university students from various countries (Torres et al., 2017; Fumaneeshoat and Fumaneeshoat, 2019; Ud Din et al., 2019; Tayfur and Evrensel, 2020). On the other hand, divergent findings have also been presented in relation to gender. For example, Richter et al. (2016) identified that females had a higher prevalence compared to males among university students in Germany. Similarly, these findings were evidenced in a study conducted by Edman and Yates (2004) in a sample of 184 Malaysian universities. In addition, a meta-analysis that considered research with a university samples (145,629 participants) from 39 Western and non-Western countries found that women also reported higher scores (Alhaj et al., 2022). Although our results found no significant differences according to gender, it is important to consider that the scientific literature has reported a higher prevalence of eating disorders in women. Therefore, it is relevant to evaluate the scores of the instrument in both men and women in South American university samples.

In relation to age, our results indicated that there were no significant differences in instrument scores between different age groups in Peruvian university students. This is congruent with that reported by Sharma et al. (2019) in a sample of 245 Indian medical university students, where no significant differences were found between groups by age. In contrast, other studies have found significant differences in relation to age. For example, in Pakistan it was reported that younger university students (under 20 years of age) presented a higher prevalence of ED risk attitudes (Ud Din et al., 2019). Similarly, in a study conducted in Europe with a sample of 561 Spanish university students, a significant difference was found in relation to age (Escolar et al., 2017). Similarly, most of the existing studies in the scientific literature contradict the results obtained by indicating that younger university students have a higher risk of developing ED. This may be because they indicate greater concern and overestimation of their body image in the face of increased exposure to social networks and beauty standards (Soto Ruiz et al., 2015; Lindvall Dahlgren et al., 2017; Zila-Velasque et al., 2022).

## Limitations

One of the main limitations of this study lies in the use of non-probability sampling using the snowball technique. This implies that participants were recruited through the recommendation of other participants, which may introduce biases in sample selection. Therefore, caution should be exercised when generalizing the results of this study to the general population of university students, as the sample may not be representative of the target population. To generalize, it is suggested to use probability sampling, which allows the inclusion of all students with a known probability. An additional limitation is related to the inclusion of university students from a specific location, in this case Lima. However, as there is diversity within the Peruvian context, an evaluation with a larger number of participants from different locations is required. In addition, it is suggested that cross-cultural research is needed to determine the consistency and structure of EAT-8 in different geographical contexts of Peru. In addition, it is also relevant to conduct longitudinal studies to identify changes in EAT-8 scores after the state of social confinement because of COVID-19, given that during this period an increase in EBDs could be noted in different contexts (Cerniglia and Cimino, 2023; Napp et al., 2023). It should be noted that the present study was conducted before social isolation, which allowed us to maintain a view on EAT-8 levels prior to that period. It should be noted that EAT-8 is not a diagnostic tool that can identify any ED, instead, it is a quick measure that detects a possible risk of experiencing an ED. For this reason, when high scores are found, it is important to supplement with other instruments and conduct a diagnostic interview based on DSM-5 or ICD-11 criteria. Finally, in the current study only a single assessment measure for eating disorders (EAT-8) was administered, thus future researchers are encouraged to perform a correlation analysis with other psychological and clinical measures in order to determine concurrent validity.

## Conclusion

It is concluded that in Peruvian university students, the EAT-8 has demonstrated a solid validity, supported by its internal structure, as well as a high reliability through internal consistency, in a unidimensional model. It was also observed that the instrument is invariant in relation to sex and age, indicating that the items are understood in a similar way in both groups. In the Bayesian comparison according to sex and age, we found a higher probability of accepting the null hypothesis, indicating that no significant differences were found. These findings are relevant because they highlight the usefulness of a brief and quickly applicable instrument to assess the risk of eating disorders in the university population.

## Data availability statement

The raw data supporting the conclusions of this article will be made available by the authors, without undue reservation.

## Ethics statement

The studies involving humans were approved by Research Ethics Committee of the Universidad Autónoma del Perú. The studies were conducted in accordance with the local legislation and institutional requirements. The participants provided their written informed consent to participate in this study.

## Author contributions

CR-V was in charge of the project as the principal investigator. MB-D and SO participated in the study design. CR-V, MB-D, and SO collaborated in the survey design, data collection, and analysis. MB-D and JS wrote the first draft of the manuscript. All authors contributed to the article and approved the submitted version.
